# Cold plasma technology: A cutting-edge approach for enhancing shrimp preservation

**DOI:** 10.1016/j.heliyon.2024.e40460

**Published:** 2024-11-15

**Authors:** Fataneh Hashempour-baltork, Adel Mirza Alizadeh, Mansoureh Taghizadeh, Hedayat Hosseini

**Affiliations:** a Halal Research Center of IRI, Iran Food and Drug Administration, Ministry of Health and Medical Education, Tehran, Iran; b Department of Food Safety and Hygiene, School of Public Health, Zanjan University of Medical Sciences, Zanjan, Iran; c Department of Nutrition, Faculty of Medicine, Mashhad University of Medical Sciences, Mashhad, Iran; d Department of Food Science and Technology, National Nutrition and Food Technology Research Institute, Faculty of Nutrition Science and Food Technology, Shahid Beheshti University of Medical Sciences, Tehran, Iran

**Keywords:** Cold plasma, Melanosis, Microbial spoilage, Quality, Sensory, Shrimp

## Abstract

Cold plasma (CP) is an emerging technology employed to safeguard highly perishable food items, particularly aquatic products such as shrimp. Due to its significant amount of moisture, superior protein composition that contains important amino acids, and unsaturated fatty acid content, shrimp are susceptible to microbial deterioration and overall alterations in their physical and chemical characteristics. Such spoilage not only diminishes the nutritional value of shrimp but also has the potential to generate harmful substances, rendering it unsuitable for consumption. Recent observations have indicated a growing market demand for shrimp that maintains its quality and has a prolonged shelf life. Furthermore, there is a significant emphasis on the production of food items that undergo minimal processing or nonthermal preservation methods. Extensive documentation exists regarding the efficacy of CP technology in eliminating microorganisms from shrimp without inducing resistance or activating enzymes that contribute to shrimp spoilage. Therefore, CP can be mentioned as a slight processing interference to preserve shrimp quality. This chapter primarily explores the principles and methods of CP technology, as well as its impact on melanosis, physicochemical changes, microbial and sensory properties, and the preservation of shrimp quality.

## 1 Introduction

Cold plasma (CP) technology has advanced significantly in the food and marine industries, providing numerous advantages in terms of safety, quality, and sustainability. This non-thermal approach has been shown improves in drying performance of food by lower energy consumption, modify the barrier properties of packaging materials to prolong the shelf life of food goods as well as degrading pesticides and allergens with reactive oxygen and nitrogen species [[1], [2], [3], [4], [5]]. Additionally, as CP techniques use smaller amounts of water, zero chemical residues, and utilize ambient atmosphere as their operating gases, they are healthier for the environment [6]. In particular, by inactivating microorganisms and triggering enzymes responsible for spoilage, CP technology helps preserve the quality and prolong the shelf life of aquatic food products [7,8].

CP is made up of a diversity of excited-state atomic, molecular, ionic, and radical species, as well as reactive species such as electric charges, positively and negatively charged ions, gaseous atomic particles liberated radicals, and stimulated molecules, as well as radiation from electromagnetic fields quanta e.g. visible light and ultraviolet rays [9]. Plasma may be categorized into high-pressure and low atmospheric plasmas. According to the characteristics governing its creation, plasma may also be categorized into thermal (high-temperature plasma) and nonthermal (low-temperature plasma) [10]. In addition, atmospheric cold plasma (ACP) and low-pressure cold plasma (LCP) are two other ways that nonthermal plasma can be produced. The usefulness of CP for usage in animal products manufacturing regions where excessive heat is not desirable has been placed. As a result, CP technology is known as nonthermal technology [11]. Numerous different possible interactions between the gas utilized in plasma generation and the material through the plasma' treatment are presented such as cross-linking, etching, and deposition or grafting on substrates [12].

**Fig. 1:**
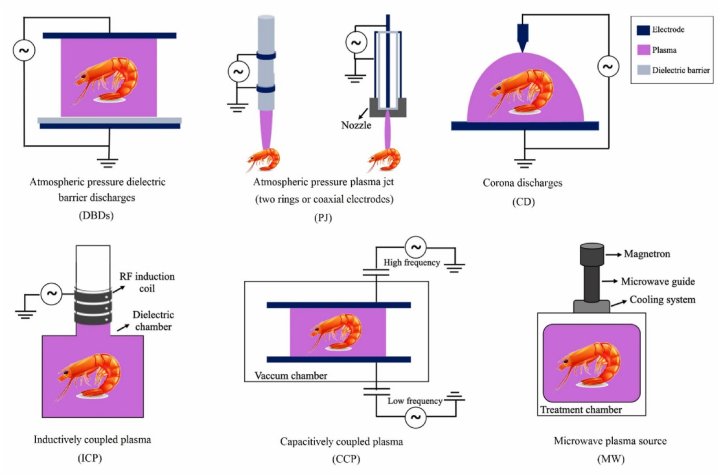
Schematic of diverse cold atmospheric pressure plasma devices (edited from Ref. [71]).

To generate CP, several different electrical discharges can be employed. Examples of these discharges include corona discharge [13], microwave discharge (MW), radiofrequency discharge [14], dielectric barrier discharges (DBDs), resistive barrier discharge system (RBDS), gliding arc (GA) discharges, and AP plasma jet (PJ) (Fig. 1) [15]. The most frequent reactive species produced in plasma processing are those containing oxygen, such as ozone (O_3_), atomic oxygen (O), excited oxygen (O_2_), singlet oxygen (^1^O_2_), and superoxide anion (O_2_^−^), and nitrogen, such as nitric oxide (NO^●^), excited nitrogen (N_2_), and atomic nitrogen (N) species. RNS detected in humid air plasma (ROONO) include nitric oxide (NO^●^), nitrogen dioxide radical (^●^NO2), peroxynitrite (ONOO-), peroxynitrous acid (OONOH), and alkylperoxynitrite [16].

The spread application of CP in the food industry has been considered in recent decade. Research regarding the agricultural products and processed food has been studied with the objective of decontamination (microbial, viruses, toxins, parasites, and etc.), particularly caused by non-thermal, cost-effective, versatile, and environmentally friendly [17,18]. It consists of various chemical species, including ions and metastable molecules, that interact with bio-surfaces. Additionally, its ultraviolet radiation aids in the decontamination process [10]. Furthermore, CP has been utilized to increase the surface energy of polymers used in food packaging, improving adherence and printability [19,20]. This makes it an operative technique for decontaminating both food items and packaging materials [21]. According to the best of our knowledge for CP application on shrimps, its effects on the overall quality characteristic of shrimp are not well reviewed. To fill this gap, this review conducted to provide a literature overview with focus on the effects of CP on the microbiological and physicochemical properties (WHC, texture, color, lipid oxidation), melanosis, sensory attributes beside the effect on nutritional value of different shrimps and also CP technology limitations [22].

## 2 Shrimp treatment with CP

Shrimp is a valuable food resource renowned for its nutritional value and strong demand [23]. However, shrimp are highly perishable and begin to deteriorate within a few hours after being harvested at room temperature [7]. The swift deterioration of shrimp is primarily caused by the rapid onset of biochemical and microbial activity following the shrimp's death [24]. According to Gonçalves and de Oliveira (2016), the quality of shrimp can decline at various stages of processing, including during frozen storage, thawing, and fluctuations in temperature [25]. Deleterious phenomena such as “lipid oxidation, protein degradation, and the formation of black spot” have been identified as the key culprits responsible for the deterioration of shrimp quality. In a recent incident, China reported the presence of COVID-19 in frozen white shrimp imported from Ecuador, highlighting the need for technologies and additives to confirm seafood safety and prolong shelf life without significantly altering its sensory properties [26]. Thus, it is crucial to develop suitable preservation approaches and additives to safeguard the quality and extend the shelf life of seafood products [27,28].

Emerging nonthermal technologies offer a delicate balance between ensuring food safety and minimizing processing steps, considering economic limitations while maintaining acceptable quality standards, and bridging the gap between innovative approaches and traditional processing methods [29]. These novel approaches have the potential to effectively deactivate microorganisms in food, ensuring food safety without relying on heat application while also preserving the nutritional value of the treated food [30]. Among these developing technologies, CP has garnered significant attention for its diverse applications in the food production.

CP technology has experienced rapid advancement as an innovative nonthermal approach with diverse uses, such as microbial sterilization, cancer therapy, and wound healing [31]. In recent times, CP technology has been employed for preserving various food items, such as fresh farmed produce [32], fish [33,34], meat [35], and shrimp [36]. The predominant focus of the existing literature has been on evaluating the antimicrobial efficacy of CP. This is achieved through the generation of various species, including “reactive nitrogen species” (RNS) and “reactive oxygen species” (ROS), such as ozone, peroxide, singlet oxygen, and different types of nitrogen oxides. The presence of these species is integral to the remarkable capacity of CP to effectively hinder bacterial growth [35].

## 3 Microbial

Hu et al., 2023 [37], explored the mechanism behind the inactivation of *Shewanella* (S.) *putrefaciens* using ACP in both phosphate-buffered saline (PBS) and sterile shrimp juice. The findings demonstrated a significant bactericidal impact of DBD-ACP treatment on *S. putrefaciens*. The primary cause of bacterial inactivation was identified as the synergistic action of induced ROS and nitrogen oxides. The utilization of ACP significantly damaged S. putrefaciens's membrane and cell wall, impairing their structure and function and causing a large intracellular material leak (such as AKP, nucleic acids, and proteins). Additionally, DNA damage was demonstrated, and the ability of *S. putrefaciens* cells to proliferate was lost. Interestingly, the cyclic treatment method, which subjected *S. putrefaciens* to prolonged exposure to reactive oxygen species (ROS), exhibited a stronger bactericidal effect rather than the one-time treatment method.

Furthermore, this mode resulted in increased levels of O3 and nitrogen oxides. However, a protecting effect regarding ACP treatment was noted in the sterile shrimp juice medium, presenting a challenge for the practical application of ACP sterilization and emphasizing the importance of enhancing ACP treatment effectiveness, through the implementation of the cyclic treatment method. In conclusion, the findings indicate that the ACP cyclic treatment mode exhibits significant prospective for surface sterilization and prolonging the aquatic products shelf life [37].

[38] indicated the capability of CP to impede bacterial growth and enzymatic activity, which contribute to the production of alkaline compounds that lead to pH decreasing in white shrimp. Likewise [36], suggested that CP did not directly affect the pH increase but effectively controlled the rate of increase by reducing microbial contamination and inactivating enzymes. In a recent investigation, researchers examined the changes in microbiological properties in storage time using conventional tap water ice and PAW ice. Notably, PAW ice exhibited an important benefit over tap water ice by inhibiting microbial growth, thereby prolonging the storage period by 4–8 days [24].

[36] evaluated the combination of CP with CLE (1 %) in Pacific white shrimp. Their research leading to a notable inhibition of various microbial counts including total viable count, psychrotrophic bacterial count, *Pseudomonas* count, hydrogen sulfide-producing bacteria, and Enterobacteriaceae count. The presence of the natural plant extract caused cell wall permeabilization, which lead to additional enhanced the antimicrobial properties of CP. Specifically, samples treated with a voltage of 15 kV after 10 days of storage exhibited the lowest levels of total viable counts (4.58 log CFU g^−1^) and psychrophilic bacterial counts (5.65 log CFU g-1). This significant reduction in microbial load was attributed to irreversible electroporation and the usage of high specific energy.

**Fig. 2:**
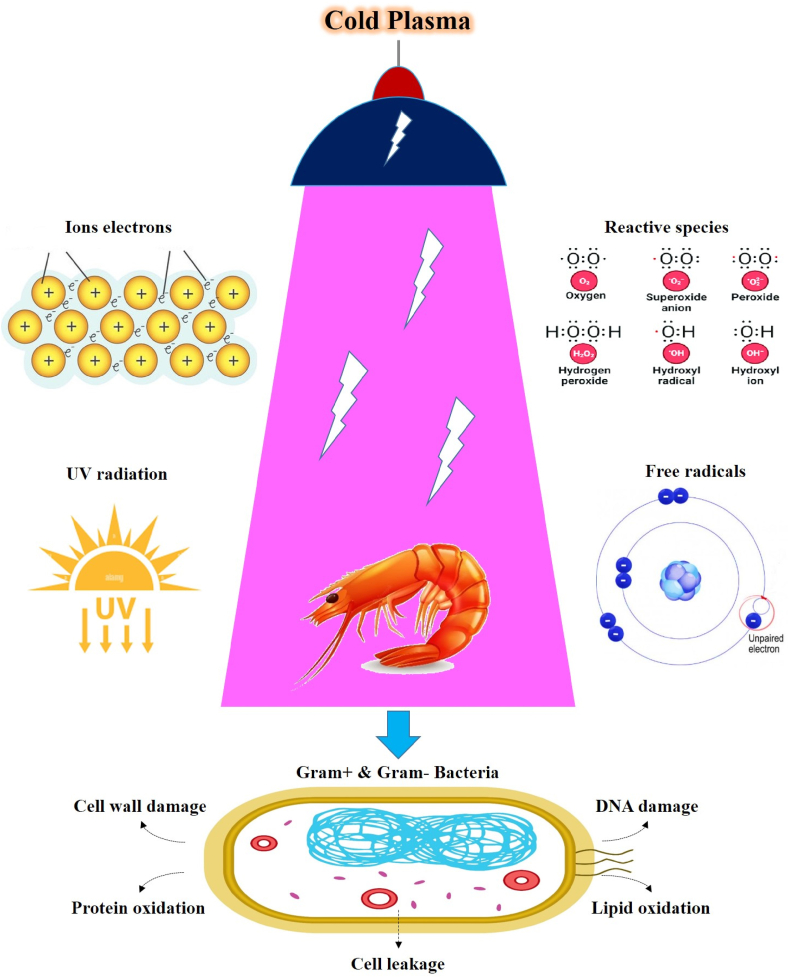
A schematic of the bactericidal mechanism of cold plasma in shrimp.

The bactericidal property of CP in its ability to damage the cellular structure of microbes on the surface of shrimp is shown in Fig. 2.

In a study conducted by Liu (2024), CP treatment for 30 min using 80 kV was found to significantly enhance the reduction of *L. monocytogenes* and *V. parahaemolyticus* in fresh shrimp (0.94 and 1.26 log CFU/g) and frozen shrimp (1.68 and 2.0 log CFU/g). During storage at 4 °C, CP-treated shrimps showed higher levels of thiobarbituric acid. Overall, these shrimps showed lower microbial counts, reduced loss of total nitrogen, minimal changes in pH, and improved quality characteristics. Furthermore, CP treatment extended the shelf life of shrimp by 2 days [39]. In Wu's (2023) research, the impact of cold atmospheric plasma pretreatment on the quality of prepared red shrimp during refrigerator storage was examined. The findings revealed that CAP pretreatment significantly decreased the total viable count, total volatile base nitrogen content (TVB-N), and polyphenol oxidase (PPO) activity in comparison to the control treatment [40]. A new method was employed to test the effectiveness of combining cold atmospheric plasma and chitosan oligosaccharide [41] treatment in enhancing the antibacterial activity and prolonging the shelf life of Pacific white shrimp (*Litopenaeus vannamei*). The findings demonstrated that the combination of COS and CAP effectively inhibited the growth of microorganisms. The treated groups exhibited lower levels of total volatile base nitrogen (TVB-N), total viable count, and pH compared to the control group [42].

## 4 Melanosis

Melanosis is a significant factor contributing to the degradation of crustaceans, leading to substantial losses in the shrimp industry. The process involves a progressive darkening of the shellfish's joints and exoskeleton, particularly in the cephalothorax region, resulting from the enzymatic oxidation of phenolic compounds. Although melanosis does not pose any direct harm to consumers and is not explicitly linked to microbial spoilage, it negatively impacts sensory attributes, resulting in a reduction in both the shelf-life and the overall product quality [25].

The CP technology induces changes in the structure of enzymes, thereby altering their functionality through the action of reactive species caused by CP [10,43]. A study conducted by de Souza Silva et al. [38] examined the potential mechanism of CP in inhibiting polyphenol oxidase in white shrimp. The active species produced by CP initiate the oxidation of aromatic amino acids found in polyphenol oxidase, leading to modifications in protein structure through interactions between amino acid side chains and the reactive species. This interaction inhibits the enzyme's activity, thus delaying melanosis. Similarly, Koddy et al. [44] stated a 50 % reduction in proteolytic activity following a 240-s CP treatment. The duration of CP exposure was found to affect protease activity, with squid mantle protein concentrate exhibiting reduced proteolytic activity as the CP exposure time increased [45].

In a research investigation, the usage of plasma-activated water (PAW) ice for shrimp conservation did not result in significant differences in lightness (L∗) and yellow/blue (b∗) values compared to tap water ice. However, the reactive species produced by CP were effective in reducing melanosis and polyphenol oxidase activity in shrimp. As a result, the redness value (a∗) of the shrimp could be maintained for up to 7 days when preserved with PAW ice, whereas tap water ice only preserved the redness value for 4 days [24].

Furthermore, the researchers [38] demonstrated that melanosis inhibition was particularly notable during storage at 5 °C following CP treatment at a frequency of 500 Hz for 10 min by using air as the working gas composition. Additionally, the use of 0.5 or 1 % Chamuang leaf extract (CLE) as a pretreatment, followed by CP treatment produced at 16 kVRMS for 10 min by different gas combinations (Ar and O2, 80:20) or (Ar and zero air), significantly reduced melanosis in pacific white shrimp during storage at 4 °C [36].

[46], had been detected that exposure to CP technology effectively reduced the polyphenol oxidase enzyme activity. Notably, samples treated for 90 s and 150 s exhibited significantly lower levels of melanosis. Extending the CP exposure to 150 s lead to a 50 % lessening in enzyme activity. According the results, CP treatment duration of 90 s was the most effective approach to prolong the shelf life of shrimp during storage [46].

Furthermore [36], had been suggested that the mixture of high voltage cold atmospheric plasma (HV-CAP) and 1 % Chamuang leaf extract (CLE) as a nonthermal treatment could extend the shelf life of shrimp. The combined impact of HV-CAP and CLE resulted in negligible levels of melanosis in the treated samples.

## 5 Physical and chemical

The acceptability of a particular food is determined by its physicochemical qualities, including factors such as color, texture, water holding capacity (WHC), lipid and protein oxidation, and overall changes. In shrimp, the application of CP technology can have various physicochemical effects, which are described as follows.

### 5.1 Color

The acceptability of seafood is significantly influenced by its coloration. However, the reactive species produced during CP treatment can induce several physicochemical changes in seafood, while if these changes are not managed, it could create difficulties when implementing this technology. One such reactive species is ozone, which is produced when a gas mixture comprising oxygen is applied in CP treatment. The oxidation potential of ozone is recorded as −2.07 V [47] and can initiate various reactions that negatively impact meat quality, particularly its color. In meat, the pigment responsible for redness is oxymyoglobin [48]. During the oxidation process, oxymyoglobin can undergo a transformation into metmyoglobin, which exhibits a brownish hue [47,49].

Literature have documented the effects of plasma on the color features, specifically “lightness (L∗), redness (a∗), and yellowness (b∗)”, of seafood (such as fish and shrimp), considering the working gas mixture and voltage parameters. Researchers have observed that samples exposed to CP generated using gas mixtures of Ar and O2 (90 % and 10 %) as gas A or CO2, Ar and O2 (60 %, 30 % and 10 %) as gas B for 5 min exhibited rises in L∗ and b∗ values, along with a reduction in a∗ value. The variations in color characteristics were more noticeable in samples subjected to gas A compared to those treated with gas B. This disparity can be attributed to the elevated ozone concentration present in gas A [50].

The duration of CP treatment is a significant factor affect the color of seafood subjected to CP. This could be attributed to the high levels of ROS and RNS with longer treatment times [34]. Choi et al. [51] also observed an increase in ΔE, indicating the level of color change, with longer CP exposure times for dried seafood. The L∗ and b∗ values of the samples increased, while the a∗ value lessened as the treatment duration with CP produced using a mixture of Ar and O2 (90 % and 10 %) was prolonged [34,49]. Therefore, based on the observations, it can be deduced that incorporating oxygen as a component gas in plasma treatment could change the color of seafood. Moreover, the specific process parameters of CP had a substantial impact on the extent of color modifications in the treated seafood.

### 5.2 Texture

Studies have reported that CP technology can preserve the textural qualities, specifically shear force, of seafood during storage. For instance Ref. [38], demonstrated that CP treatment helped maintain the textural values of seafood for up to 12 days of storage. The exposure to CP lead to the development of denser proteins, which enhanced the textural qualities of the seafood [45]. In the case of shrimp [24], found that using of ice formed by PAW helped retain the firmness of shrimp. This preservation effect was attributed to the inhibition of microbial and enzymatic degradation by CP. Furthermore, no notable difference in the hardness level was indicated in sea bream fillets following treatment with plasma [52].

### 5.3 WHC

WHC pertains to the meat's capacity to retain water when exposed to external pressure, thereby directly influencing its moisture content. Also, it can be characterized as the meat's ability to retain additional water. The slight rise in WHC noticed in samples treated with CP was ascribed to the greater surface area exposed to CP energy and longer treatment durations, leading to enhanced water retention. It is known that plasma can cause etching on surfaces [53]. However, the complete understanding of how CP affects the WHC in seafood remains unknown. Though, the alteration of proteins in seafood caused by the reactive substances produced by plasma might impact the WHC of seafood that has been treated. A recent study [38] described that pacific white shrimp preserved with CP produced by using air as the working gas composition for 10 min exhibited a higher WHC rather than the untreated control samples. This trend was also observed during the storage of shrimp at low temperatures, specifically at 5 °C, for a duration of 12 days [38]. Consequently, more investigation is needed to fully understand how plasma affects seafood's WHC because higher WHC is a desired quality linked to juiciness and sensitive texture.

### 5.4 Lipid oxidation

Variations in lipid content, composition, and improper handling can contribute to increased lipid oxidation. It should be emphasized that the orientation of the sample during plasma treatment, with the skin facing downward, could also account for differences in lipid oxidation. However, according to existing literature, it is recommended to follow an optimized procedure when applying plasma in order to minimize lipid oxidation. Likewise, the utilization of ice produced from PAW has negligible effects on lipid oxidation in shrimp and can be disregarded [24]. PAW ice has the ability to prevent microbial growth and enzymatic activity and slow the oxidation of lipid during storage. Additionally, the application of CP treatment did not exhibit any significant impact on oxidation of lipid in mackerel, gaining results comparable to untreated samples [54].

A research study discovered that the oxidation of lipids caused by CP lead to a reduction in the presence of monounsaturated fatty acids (MUFAs) and polyunsaturated fatty acids (PUFAs), while simultaneously increasing the levels of saturated fatty acids (SFAs) [50]. However, pretreatment with natural extracts, such as coconut husk extract in either free or encapsulated form, followed by CP treatment, was found to mitigate the degradation of MUFAs and PUFAs caused by the antioxidant activity of the natural extracts.

These findings indicate that employing natural extracts possessing antioxidant properties can be beneficial in preventing the depletion of MUFAs and PUFAs caused by CP. Likewise, in a study directed by Ref. [33], it was observed that pre-treating with antioxidants reduced the oxidation of vulnerable MUFAs and PUFAs, consequently leading to the formation of SFAs. Though, samples treated with ethanolic coconut husk extract exhibited a significantly slower rate of lessening compared to samples treated with ascorbic acid. In other study, variations in the fatty acid composition were observed in Pacific white shrimp treated with coconut husk extract, CP and packaged under different gas compositions [36].

Omega-3 fatty acids exhibited the least oxidation in the presence of the gas composition with 80 % argon and 20 % air, primarily due to the lower levels of oxygen present, and pretreatment with CHE further reduced the loss of PUFAs and MUFAs. This suggests that pretreatment with natural extracts and modification of the gas mixture under plasma treatment can mitigate the loss of PUFAs and MUFAs [36]. indicated that HV-CAP combined with CLE was a proper non-thermal treatment for extending the shrimp shelf-life. The inclusion of antioxidants, especially CLE, significantly reduced the oxidation of lipid when HV-CAP was applied. Shrimps treated with HV-CAP, particularly the samples which treated with 1 % CLE with high antioxidant capability, exhibited lower levels oxidation in lipid and protein during storage.

### 5.5 Overall properties

Several studies have demonstrated the success of CP in inhibiting various enzymes. A research study by Ref. [38] suggested that CP treatment could inhibit polyphenol oxidase, thereby prolonging the shelf life of white shrimp. Additionally, numerous studies have described that CP treatment leads to reduction in protein degradation, protein oxidation, protein solubility, sulfhydryl content of proteins, as well as lipid oxidation. Conversely, the application of CP treatment has been linked to various effects, including heightened protein aggregation, increased carbonyl content in proteins, altered surface hydrophobicity and gelling characteristics of myofibrillar proteins. These effects are thought to be attributed to the prevention of lipases, proteases, and other related enzymes caused by CP treatment [33,36,55]. Ice formed from PAW has also been shown to slow down the pH increase in shrimp rather than tap water ice [24]. Though, the marginal rise in pH can be ascribed to decreased protein degradation, leading to reduced levels of alkaline compounds that are responsible for pH fluctuations. The study revealed that shrimp subjected to CP treatment along with metabisulfite demonstrated a slower rate of pH increase, total volatile base nitrogen content, thiobarbituric acid reactive substances, free fatty acids, peroxide value, and fluorescent compounds rather than the control group (P < 0.05). While CP treatment reduced biochemical alterations in shrimp treated for 45 s and 150 s, the optimal treatment for minimizing unfavorable changes and enhancing shrimp quality was observed at 90 s [46].

In other study, the researchers examined the special effects of conventional tap water ice and PAW ice on the physicochemical, and protein properties of stored shrimp. Throughout the storage duration, shrimps following treating with PAW ice consistently kept a pH level under 7.7. In addition, the samples treated with PAW ice, experienced the delayed deterioration in color and hardness attributes. The generation of volatile basic nitrogen was significantly lessened to below 20 mg/100 g in the PAW ice storage, which was notably lesser (p < 0.05) compared to the shrimps which treated with regular tap water ice. Importantly, the utilization of PAW ice did not lead to any unfavorable alterations in shrimp proteins [24].

In an investigation conducted by Ref. [56], researchers examined how DBD CP impacted the structure, hydrophobicity on the surface, and allergenic characteristics of shrimp tropomyosin. The findings demonstrated that, increasing in duration of CP treatment, lead to rise in the molecular weight of tropomyosin and decrease in the protein concentration. Additionally, the amount of free amino acids rose by 74.7 %, and there were changes in the spreading of aromatic amino acids. After undergoing a 20-min treatment, the α-helix content experienced a reduction of 69 %, while the surface hydrophobicity enhanced by 57.8 %. The analysis of allergenicity revealed a significant reduction of 96 % in binding capacity of IgE following the 20-min treatment. Furthermore, the degranulation indexes of KU812 cells, including the release rate of β-HEX, intensity of intracellular calcium ions, release of histamine, and levels of IL-4, TNF-α (as inflammatory cytokines), exhibited reductions of 32.5, 31.0, 37.3, 51.7, and 70.2 %, respectively. Thus, this investigation provides confirmation that DBD CP can effectively diminish the allergenicity of tropomyosin through structural modifications.

Furthermore [57], conducted a study on the biological analysis of CP's effect on reducing the allergenicity of tropomyosin in shrimp. They investigated the in vivo biological regulation of tropomyosin allergenicity using a mouse model. Treatment with CP led to a decrease in allergic symptoms in mice and helped maintain the balance between Th1 and Th2 immune responses to avoid allergies. This effect was observed through the activation of Treg cells, as indicated by serum and cytokine analysis. Moreover, examination of the gut flora revealed a connection between the prevalence of allergies and a rise in the species variety and a fall in the species abundance of the gut microbiota. Significantly, the group subjected to tropomyosin and the control group administered with PBS differed significantly in terms of species composition, pointing to a possible link between bacterial variety and allergies. Certain bacterial groups, such as Firmicutes, Bacteroidetes, Parabacteroides, Alloprevotella, Bacteroides, and Lachnospiraceae, were found to be associated with allergy occurrence. Analysis of intestinal sections indicated that occurrence of allergy was accompanied by intestinal structural damage, which CP treatment was able to alleviate.

In a study assessing the CP impact on the quality properties of Pacific white shrimp (*Litopenaeus vannamei*), it was observed that samples treated with nonthermal plasma for 10 min at a frequency of 500 Hz showed an extended shelf life of 14.07 days, whereas the control sample had a shelf life of 9.78 days [38]. The literature review indicated that CP treatment of shrimp could improve the physicochemical properties, including color, texture, protein and lipid oxidation, in addition to the reduction of melanosis and allergenicity during storage, thereby prolonging the shelf life.

## 6 Sensory

The sensory perception of food plays a crucial role in determining its acceptability to consumers. Various reactions, especially lipid oxidation, can have an undesirable effect on the sensory attributes of seafood treated with CP. Thus, it is essential to carefully select proper process parameters that ensure the seafood safety and extend its shelf life while minimizing any adverse effects on sensory properties. Due to the high fat content in seafood, the reactive species generated by CP can directly induce the formation of lipid oxidation products, which can result in off-flavors or off-odors in samples which reared with CP [58,59]. These oxidation products, mainly secondary products, distinct as "fishy," "rancid", "metallic", or "oxidized". The fishy odor, in particular, is a main attribute that can lead to rejection and can be caused by the reactive species generated by CP [34,49].

According to a study by Ref. [60], 2,4-heptadienal and hexanal were identified as predominant aldehydes in fish and seafood oil. These aldehydes, along with others like heptanal and nonanal, are formed through lipid oxidation processes. Also, these compounds, along with others like 1-penten-3-one and cis-4-heptenal, are formed through lipid oxidation processes during storage and processing [61] The development of fishy odor compounds is affected by various factors, including environmental conditions, diet, enzymatic reactions, and microbial metabolism [61].

At lower power and shorter treatment times, CP has shown minimal impacts on physical, chemical, nutritional, and sensory attributes of foods [62]. However, higher intensities and longer treatments can significantly affect carbohydrates, lipids, and proteins [62]. CP can modify protein structure, induce lipid oxidation, and alter starch properties [63]. While CP shows promise in various food applications, challenges remain in scaling up the technology and ensuring uniform treatment [64]. Further research is needed to optimize CP treatments and fully understand its effects on food components at the molecular level.

In a study conducted by by Ref. [65], 1-octen-3-ol and hexanal were identified as the main volatile compounds lead to the fishy odor in samples which storing at low-temperature. The sensory characteristics such as flavor, odor, taste, and overall likeness scores of seafood samples varied dependent on the duration of CP treatment, but generally scored lesser than the control group, mainly with more treatment times of 7.5 and 10 min [34,49].

Elevated levels of thiobarbituric acid reactive substances (TBARS) were found to be correlated with the changes observed in sensory attributes of the samples, which can lead to undesirable tastes and odors, ultimately rendering the seafood unacceptable to consumers [34,49]. thus, the sensory characteristics of CP-treated seafood are significantly influenced, especially in severe conditions or while high concentrations of active species are generated. This is primarily attributed to accelerated lipid oxidation in seafood.

It's worth noting that there are limited studies highlighting substantial alterations in the sensory qualities of shrimp following CP treatment.

For example, in a research study by Ref. [38], it was detected that the control groups of white shrimp (*Litopenaeus vannamei*) had superior sensory quality compared to CP-treated samples. Similarly, in other investigation, Asian sea bass slices subjected to CP treatment exhibited undesirable tastes and odors [50]. The reduced sensory scores for odor were dependent to the production of volatile compounds that contributed to the off-odors. However, certain studies have indicated that pre-treatment with natural extracts, such as CHE, effectively mitigated the negative effect on sensory properties during shelf life [36,50].

[36] reported that pretreating Pacific white shrimp with CLE followed by CP treatment resulted in acceptable sensory scores for up to 15 days. Likewise, another study reported no adverse effect of CP treatment when chitooligosaccharide was added [66]. Therefore, despite the negative impact of CP on the sensory scores of treated shrimps, the use of different additives has the potential to improve sensory qualities. A summary of the studies conducted regarding the use of CP for shrimp treatment is given in Table 1.

**Table 1:** Application of CP in shrimp.

Shrimp	Parameters	Types	Results	Reference
Pacific white shrimp (*Litopenaeus vannamei*)	Atmospheric air 40 kV and 500 Hz for 10 min	DBD plasma	DBD plasma lead to important reduction in the microorganism inhibited Mesophilic bacteria, psychrotrophic bacteria, and *Staphylococcus* spp.	[38]
Pacific white shrimp (*Litopenaeus vannamei*)	Argon/air (80:20) 16 kVRMS for 10 min	IB-DBD plasma	In-bag dielectric barrier discharge CP (IB-DBD) of shrimp presoaked in CLE. Microorganisms inhibited: total viable counts, Lipid oxidation: reduced, Protein oxidation: reduced.	[36]
Shrimp (*Metapenaeusensis*)	Input power of 30 was used, for 10 min	DBD plasma	Microorganisms inhibited: total viable count, Lipid oxidation: increased (slight), Protein oxidation: no change.	[24]
Pacific white shrimp (*Litopenaeus vannamei*)	Air, 220 V, 282 W, 50 Hz, and 1.9 A with 45, 90, 150 s durations	Pj	Application of CP for 90 s was the most efficient circumstance to extend shelf life.	[46]
Red shrimp (*Solenocera crassicornis)*	Air, with a maximum voltage output of 35 kV at 50 Hz for 5 min	ACP	The use of ACP cyclical treatment resulted in a surface bacterial count reduction of 0.52 log CFU/mL in whole shrimps, whereas ACP one-time treatment only achieved a decrease of 0.18 log CFU/mL.	[37]
Shrimp (*Fenneropenaeus Chinensis*))	Input voltage 50 V, input current 1 A, applied voltage 50 kV) with different times (0, 5, 10, 15, and 20 min).	DBD plasma	After a 20-min treatment, the IgE binding capacity decreased by 96 %. Additionally, the degranulation indexes of KU812 cells, such as the release rate of β-HEX, the intensity of intracellular calcium ions, and the release of histamine and inflammatory cytokines (IL-4, TNF-α), were reduced by 32.5 %, 31.0 %, 37.3 %, 51.7 %, and 70.2 %, respectively.	[56]
Pacific White Shrimp (*Litopenaeus vannamei*)	50–100 % argon gas concentrations, a 2-to 10-min process duration, and a 3-to-7-centimeter distance from the sample	Gliding Arc Plasma Discharge	Sample distance had less of an impact on the PWS's TMAB, PV, ΔE, and sensory characteristics than did argon gas and treatment duration. In contrast to low quantities of argon gas (5.41 CFU/ml), a high concentration of argon gas (100 %) in the plasma resulted in a reduction in TMAB (1.2 CFU/g). Moreover, elevated argon gas concentrations result in a drop in PV, an increase in ΔE, and changes to the shrimp's sensory attributes. The findings indicate that gliding arc plasma conditions with a desirability of 76.3 % can be achieved optionally by using 94.15 % argon gas for 7.10 min at a sample distance of 6.54 cm.	[113]

## 7 Improving nutritional value and nutrient preservation

Thermal processing can destroy heat-sensitive nutrients such as vitamins C and B due to high temperatures, resulting in nutrient loss due to oxidation, thermal decomposition, and leaching into the cooking water. The amount of loss of nutrients depends on temperature, time, and the presence of oxygen and water [5]. CP technology operates at low temperatures, minimizing thermal degradation of nutrients. It avoids the direct impact of heat on the nutrients, preserving the nutritional and sensory qualities of the food, including its color, taste, and texture [63].

CP technology can extend the shelf life of food products by reducing active microorganisms and enzymes that cause spoilage and food deterioration. This not only delays the process of spoilage and destruction but also helps maintain the nutritional value and properties of the food [5]. CP treatment can change the structure of food components, enhancing factors such as increased cell wall permeability, nutrient extraction, and bioavailability from food matrices [67]. Additionally, dielectric barrier discharges resulting from CP activity can boost nutritional value and preserve nutrients in food products, facilitating improvements in biological processes and preventing nutrient degradation [68]. Literature has indicated that CP treatment can improve cell wall permeability in food, enabling better extraction of nutrients and potentially increasing nutrient absorption from the food matrix [68]. One advantage of using CP is its limited heat transfer to food products compared to thermal methods. This reduces the risk of non-enzymatic browning, protein denaturation, flavor changes, and vitamin damage [69]. The application of CP dielectric barrier (DBD) on shrimp has been found to result in significant effects. Increasing the treatment time of CP leads to a notable 74.7 % enhance in the level of free amino acids. Furthermore, it induces changes in the spreading of aromatic amino acids [56]. The combination of minimal dielectric barrier depletion (MDBD) with atmospheric CP pretreatment has been studied for its effects on the biochemical and sensory quality of fresh Pacific white shrimp (*Penaeus vannamei*) stored in the refrigerator. The study found that when the shrimp were treated with MDBD-ACP for 90 s, protein degradation was observed, while at treatment times of 120 and 150 s, the degradation was negligible. This suggests that the MDBD-ACP pretreatment for 90 s was effective. in preserving the quality of the shrimp, as it prevented significant protein degradation. Protein degradation can lead to changes in the texture and sensory properties of the shrimp, potentially reducing its overall quality [70].

Overall, employing CP as a non-thermal preservation technology in the food industry holds considerable appeal. However, further in-depth research is necessary to ensure the preservation of nutrients and vitamins in shrimp.

## 8 Limitations of the use of CP in food

While CP technology has shown potential in food preservation, its current use is limited by safety, sensory, and regulatory concerns [71]. Although ozone has antimicrobial and antioxidant effects on living cells, it may be irritating. Adequate control of ozone concentration is essential to avoid any potential hazard to employees and workers [72,73]. The effectiveness of CP depends on various factors, including the type of microorganism, food matrix, and specific treatment parameters. Some of these factors may show resistance to CP treatment [5]. There are concerns about the potential formation of toxic compounds, including ozone, nitrogen oxides, and other reactive species during CP treatment. Monitoring and controlling the development of these compounds is very significant to ensure food safety [63]. While CP can maintain or enhance some food quality attributes, it may also have adverse effects. For example, CP treatment can lead to changes in the color, texture, and taste of nutritional compounds that may not match consumer preferences [5]. CP treatment is generally more effective on the surface of food products, so the limited penetration and inactivation of microorganisms in bulk or concentrated foods limits its application to certain types of food products [67].

To overcome this limitation, CP can be combined with other technologies to enhance its effectiveness. Its efficacy can be increased by joining it with other techniques, such as microwave and heat treatments [74]. Additionally, CP can be used as a pre-treatment to progress various food processes, including drying, extraction, and curing [75]. When combined with minimal processing methods, thermal and other non-thermal technologies, or antimicrobial agents, CP can increase microbial reduction efficiency and add value to the overall process.

The regulatory approval process for CP technology in food processing can be complex and varies by region [76,77]. The development of regulations for novel food processing technologies typically involves collaboration among funding agencies, research institutions, equipment suppliers, the food industry, and regulatory bodies. Therefore, the adoption of CP is confronted with a number of regulatory challenges. These encompass the demonstration of efficacy for particular products and processes, the development of scalable designs, the establishment of effective process control and validation methods, and the acquisition of regulatory approval [76,78]. The varied range of reactive species generated by CP raises questions about health and safety, as well as environmental effect [77]. As the technology progresses from laboratory to industrial scale, regulatory status becomes increasingly important for both end-users and equipment manufacturers [76]. While CP has shown possibility in numerous food processing applications, such as reducing drying time and increasing extraction efficiency, challenges remain in scaling up the technology and optimizing its effects on nutritional and sensory qualities [75].

In addition, consumer acceptance of CP-treated foods may be influenced by perceptions of safety and naturalness. That makes educating consumers and obtaining regulatory approvals critical for wider adoption of CP technology [5]. Consumer concerns about safety and naturalness may hinder its wider acceptance. Studies have shown that consumers value the perceived naturalness and improved taste of products treated with novel technologies, but they also express concerns about potential health impacts and lack of information [79]. Factors influencing consumer acceptance include food neophobia, disgust sensitivity, and cultural values [80]. Similar challenges have been observed by the other food preservation technologies, e.g. irradiation, where consumer misunderstanding and lack of acceptance have impeded widespread adoption despite its proven safety and efficacy [81].

And economically, the initial setup and operating costs of CP equipment can be high and a barrier for small and medium-sized companies. Furthermore, the integration of CP technology into existing food processing lines requires careful planning and optimization to ensure efficiency and cost-effectiveness [63].

However, studies show that overcoming these barriers can lead to substantial economic and environmental gains [82]. CP technology offers significant potential for sustainable food processing and wastewater treatment, contributing to a circular economy. It provides an energy-efficient, eco-friendly alternative to traditional approaches, reducing the usage of harmful chemicals and improving resource efficacy [77,83]. As an environmentally friendly process, CP uses minimal substances and produces little effluent, potentially providing economic advantages in industrial applications [84].

## 9 Comparison of CP with other non-thermal storage methods of shrimp

Non-thermal storage methods offer promising alternatives for preserving shrimp quality during storage. CP treatment has shown effectiveness in reducing microbial counts and maintaining quality attributes in shrimp throughout cold storage [85]. Other non-thermal processing methods, eg. irradiation, magnetic/electric fields, ultrahigh pressure, and ultrasound, have demonstrated potential in inactivating microorganisms and enhancing microbiological safety, physicochemical properties, and shelf life of aquatic products [86].

HHP treatment (250 MPa, 50 °C, 10 min) can extend shrimp shelf life to 16 days at 4 °C, compared to 4 days for unprocessed samples [87]. HHP inactivates microorganisms and enzymes, but may increase hardness and cause slight pinkness at high pressures [88]. CP treatment (15 kHz, 10 min) can extend shrimp shelf life by 5 days, delaying melanization and microbial growth while improving physicochemical and sensory qualities [89]. CP exposure for 90 s is most effective in reducing biochemical changes and melanosis, decreasing polyphenol oxidase activity by up to 50 % [46]. Both methods show promise for shrimp preservation, with CP potentially offering advantages in maintaining texture and appearance compared to HHP.

The application of pulsed electric field (PEF) treatment has demonstrated efficacy in maintaining the quality of Pacific white shrimp during cold storage. PEF treatment at 483 kJ/kg with 600 pulses reduced melanosis, retarded quality deterioration, and improved sensory scores in shrimp after 10 days of storage [90]. When combined with Chamuang leaf extract (CLE) soaking, PEF-treated shrimp exhibited lower microbial counts and oxidation markers [91]. Further enhancement was achieved by packaging PEF-CLE treated shrimp under modified atmosphere, particularly CO2, which significantly extended shelf-life [92]. Additionally, PEF pretreatment improved lipid extraction yield from shrimp cephalothorax using ultrasound-assisted extraction, while maintaining higher levels of PUFA and carotenoids [93]. These studies demonstrate that PEF treatment, especially when combined with other preservation techniques, is an effective method for maintaining shrimp quality and extending shelf-life during refrigerated storage.

Ohmic heating (OH) is an innovative food processing technique that uses electric current to heat food volumetrically and efficiently [94]. It offers advantages over traditional heating methods, including faster heating rates and better preservation of nutritional and sensory qualities [95]. OH has shown potential in various applications such as enzyme and microbial inactivation, blanching, and pasteurization [94,95]. In shrimp processing, OH has been investigated for shell loosening, but its effectiveness is limited and can even decrease peelability at higher temperatures due to collagen gelatinization [96]. However, OH has demonstrated significant benefits in other seafood applications, such as improving gel functionality in Pacific whiting surimi by minimizing protein degradation and creating a continuous network structure [97]. The effectiveness of OH can be influenced by factors like electric field strength, frequency, and food composition [94].

Microwave-assisted induction heating (MAIH) is the other developing technology for shrimp processing that couples microwave and induction heating [98]. MAIH effectively inhibits microbial growth, delays color and texture degradation, and extends the refrigerated shelf life of prepackaged shrimp rather than traditional boiling methods [98]. Optimal heating conditions for MAIH are 130 °C for 80–100s or 90 °C for 100–130s, resulting in fully cooked shrimp with desirable appearance and no detectable aerobic plate count [98,99]. MAIH allows for in-package heating and sterilization, eliminating post-contamination issues [100]. Additionally, modified atmosphere packaging (MAP) combined with green tea extract treatment can further retard quality changes in refrigerated shrimp by reducing microbial growth, chemical changes, and melanosis formation [101].

As well as, ultrasonication has shown promising applications in improving shrimp storage and processing. High-intensity ultrasound can increase the functional characteristics of shrimp myofibrillar proteins, leading to improved gelling and emulsifying characteristics [102]. This technique also facilitates the extraction of bioactive compounds from shrimp waste and addresses safety concerns by reducing allergens and inactivating pathogens [103]. Ultrasonication has been effective in encapsulating shrimp oil in nanoliposomes, preventing oxidation and masking undesirable odors during storage [104]. Additionally, ultrasound-treated fish myofibrillar protein has demonstrated enhanced emulsifying properties, stabilizing shrimp oil-in-water emulsions for extended periods [105]. While ultrasonication shows potential for improving shrimp quality and storage, further research is needed to optimize its application and address potential drawbacks, such as lipid oxidation, which may affect texture properties [103].

Low-dose irradiation (up to 6 kGy) can eliminate spoilage microorganisms without significantly affecting sensory quality or texture [106]. Higher doses (up to 7 kGy) combined with modified atmosphere packaging can further extend shelf life by reducing microbial growth and chemical degradation [107]. Irradiation induces the formation of stable free radicals in shrimp shells, which can be detected using electron spin resonance spectroscopy. While irradiation shows promise for shrimp preservation and sterilization, further research is needed to optimize dosage and assess long-term effects on shrimp quality.

Besides, literature have discovered the effects of magnetic fields on shrimp maintenance and quality. Exposure to extremely low frequency (ELF) magnetic fields can prevent pathogenic bacteria proliferation and prolong the shelf life of vannamei shrimp [108]. Static magnetic field-assisted freezing, particularly at 2 mT intensity, can significantly improve the freezing process and quality indicators of Penaeus vannamei, reducing enzyme activities, drip loss, and bacterial counts [109]. Additionally, ELF magnetic field exposure has been found to affect the pH of vannamei shrimp, with intensities of 300 μT and 500 μT showing notable impacts [110].

## 10 Future prospects

The upcoming application of CP in the food industry is promising, recent papers show its potential for wider industrial use. CP is renowned for its exceptional capability to deactivate microbes in food processing, making it a formidable tool for innovation. Additionally, CP offers benefits such as reliable performance at cold temperatures, minimal impact on the product medium, and reduced resource consumption. Increasingly, CP is acknowledged as a dependable non-thermal technique to enhance food safety without compromising food quality [111]. The growing demand for minimally processed foods has prompted the adoption of CP technology as an alternative to heat treatment. CP is an innovative and eco-friendly technology that preserves the organoleptic characteristics of food without alteration [2]. The cost-effectiveness and efficiency of ACP drive research into its potential in the food industry. ACP offers the ability to deactivate food pathogens, enzymes, food allergens, and pesticides. By adjusting its limitations, ACP can also be utilized in food modification, pretreatment drying, nutrient extraction, active packaging, and food waste processing [112]. CP has garnered attention in the meat industry for its potential to augment the microbiological safety of meat and its products. This technology has demonstrated a considerable impact on microbial control while causing minimal alterations to the physicochemical properties [113]. As a result, CP holds substantial promise as a novel treatment option for cooked meat products in the future [114]. Additionally, CP finds application in the food industry for surface disinfection of food and packaging materials. This technology provides a harmonious blend of economic considerations and superior quality, all while ensuring food stability and minimizing processing steps [21].

Consumer awareness and acceptance of shrimp products are influenced by various factors. While consumers struggle to differentiate between shrimp varieties or growth conditions, they consider flavor as the dominant factor affecting consumption [115]. Price sensitivity plays a crucial role in consumer acceptance, with studies showing that consumers may favor chemical-free crustacean products despite a price rise of up to 15 % [116]. However, acceptance of novel processing technologies, such as high-pressure processing, depends on perceived benefits and risks, including taste, convenience, nutritional value, and safety [117]. In buying situations, perceived price positively affects expected eating quality for highly involved consumers, while in usage situations, expected naturalness strongly influences experienced naturalness for the same group [118].

## 11 Conclusion

The demand for aquatic fish products that are safe, nutritious, and of high quality is increasing. The innovative nonthermal technology known as CP has been discussed as a means of preserving and ensuring the safety of aquatic food products, including shrimp. CP has been shown to successfully deactivate particular spoilage and pathogenic bacteria, such as *Enterobacteriaceae*, *P. fluorescens*, *L. monocytogenes*, *S. aureus*, *B. cereus*, hydrogen sulfite-producing bacteria, and lactic acid bacteria. This outcome is ascribed to the production of reactive species through CP, which is contingent upon factors such as the gas composition utilized, the energy quantity applied, and the duration of the treatment. Despite its remarkable ability to deactivate microorganisms, CP has been found to initiate lipid and protein oxidation, which limits its application. However, this oxidation effect can be managed by optimizing the specific conditions of CP and utilizing natural compounds that have synergistic effects. The incorporation of natural compounds aids in minimizing the deterioration due to the reactive species generated by CP. Furthermore, recent advancements have showcased a greater comprehension of the synergistic utilization of CP with various natural compositions as a "Hurdle technology" for shrimps, yielding favorable results. Besides, CP offers benefits and can be joined with the other nonthermal technologies after optimizing the process to enhance microbial destruction while preserving the nutritional value of the products.

## CRediT authorship contribution statement

**Fataneh Hashempour-baltork:** Writing – review & editing, Writing – original draft, Software, Methodology, Investigation, Data curation. **Adel Mirza Alizadeh:** Writing – review & editing, Writing – original draft, Software, Methodology, Investigation, Data curation. **Mansoureh Taghizadeh:** Writing – review & editing, Writing – original draft, Software, Methodology, Investigation, Data curation. **Hedayat Hosseini:** Writing – review & editing, Writing – original draft, Software, Methodology, Investigation, Data curation.

## Ethical considerations

There were no ethical considerations to be considered in this research.

## Data availability statement

No data was used for the research described in the article.

## Funding

This research received no external funding.

## Declaration of competing interest

The authors declare that they have no known competing financial interests or personal relationships that could have appeared to influence the work reported in this paper.
